# Direct cost does not impact on young children’s spontaneous helping behavior

**DOI:** 10.3389/fpsyg.2014.01509

**Published:** 2014-12-23

**Authors:** Mark Nielsen, Julia Gigante, Emma Collier-Baker

**Affiliations:** ^1^Early Cognitive Development Centre, School of Psychology, The University of QueenslandBrisbane, QLD, Australia; ^2^School of Applied Human Sciences, University of KwaZulu-NatalDurban, South Africa

**Keywords:** altruism, helping behavior, preschool children, prosocial behavior, social development

## Abstract

The propensity of humans to engage in prosocial behavior is unlike that of any other species. Individuals will help others even when it comes at a cost to themselves, and even when the others are complete strangers. However, to date, scant empirical evidence has been forthcoming on young children’s altruistic tendencies. To investigate this 45 4-year-olds were presented with a task in which they had opportunity to help an adult confederate retrieve a reward from a novel box. In a control condition children were given no information about the effect of potential helping behavior. Alternatively they were informed that helping would either cost them (i.e., they would miss out on getting the reward) or benefit them (i.e., they would get the reward). It was hypothesized that children would be less likely, and slower, to help in the cost condition, compared to the other two conditions. This hypothesis was not supported: children across all conditions provided help at near ceiling levels.

## INTRODUCTION

Humans are highly prosocial beings; we share our food, we give gifts, we hold open doors for people, we inform others with helpful information, and we comfort those who have lost a loved one. Moreover, capable of altruistic behavior, we will help others when it incurs a considerable cost to ourselves, and even when the recipients of the help are not related to us (Trivers, 1971; Zahavi, 2003). This tendency to engage in prosocial behavior emerges early, with, for example, children in the first half of their second year directing an adult to the location of an ostensibly missing item (Liszkowski et al., 2006). But whether or not children show altruism if such prosocial behavior incurs an immediate and direct cost has not, hitherto, been firmly established.

As already alluded to, the prosocial proclivity of infants and young children has been well documented (for a recent review see Paulus, 2014). As early as 6 months of age, infants are sensitive to fairness and prefer helpful individuals over unhelpful ones (Hamlin et al., 2007; although see Scarf et al., 2012), by 12 months they begin to provide helpful information to others (Liszkowski et al., 2008) and as they move into their second year begin sharing toys with an unfamiliar adult (Schmidt and Sommerville, 2011) and same-aged peers (Hay et al., 1991). Moreover, in this period infants become increasingly capable of providing instrumental help; that is, helping another achieve a goal. In a landmark study Warneken and Tomasello (2006) presented 18-month-olds with a range of scenarios in which an adult needed help to achieve a goal that was not directly for the infant (e.g., the adult dropped a peg out of reach while trying to hang a towel on a line or he attempted to open a cabinet while holding a stack of magazines that prohibited him from doing so). When confronted with these situations the infants spontaneously reacted by helping the adult (e.g., by retrieving the peg for him or by opening the door) without being explicitly asked to and despite receiving no reward or praise for their actions. A follow up study found 14-month-olds could show this kind of helping behavior but only on ‘out-of-reach’ tasks such as the clothesline example noted above (Warneken and Tomasello, 2007).

Moving into childhood, Brownell et al. (2009) presented 18- and 25-month-olds infants with a task requiring them to pull one of two handles attached to a pair of trays in order to obtain a reward. Pulling one of the handles delivered a loaded tray to the child and to an adult confederate, whereas pulling the alternative handle delivered a loaded tray to the child only. The 25-month-olds children chose the prosocial option, delivering food to themselves and the adult, significantly more than the 18-month-olds, with the latter needing more verbal cues from the adult to recognize the joint goal available. House et al. (2012) used a similar design with 3- to 8-year-olds, finding high levels of prosocial behavior with the younger children performing at similar levels to the older children, suggesting that spontaneous prosocial behavior becomes firmly established through the childhood period.

Further, a more recent study by Warneken and Tomasello (2013) suggests that by 3.5 years, children will start to modify their prosocial behavior depending on the partner’s previous behavior (i.e., if the partner has cooperated with them in the past or not). However, this was only apparent in sharing but not helping situations, where in the case of the latter children performed at near ceiling levels. That is, children will share more with someone who has previously shared with them, but another’s previous helping behavior does not influence children’s current helping. Nevertheless, as with the afore-cited research, the helping task did not incur any cost to the children if they decided to help, which may explain the high levels children evidenced. Indeed, in studies where children must make a choice between a division of resources that is self-advantageous, neutral or other-advantageous (e.g., Thompson et al., 1998; Fehr et al., 2008; Paulus and Moore, 2014; Williams and Moore, 2014) older children (e.g., 7- to 8-year-olds) prefer allocations that remove advantageous or disadvantageous inequality whereas younger children (3- to 4-year-olds) behave selfishly.

Further insight into what might happen to prosocial behavior in infants and young children if there is some cost involved comes from a study by Svetlova et al. (2010) who presented 18- and 30-month-olds with a series of scenarios in which an adult confederate needed instrumental help (e.g., getting a clothes pin to continue clipping fabric to a clothes line), empathetic help (e.g., getting a blanket that belonged to the adult because she was cold) or altruistic help (e.g., giving a blanket to the experimenter that belonged to the child). The latter was considered costly for the child, as he/she had to sacrifice his/her own belongings (albeit temporarily). Although 30-month-olds helped significantly more overall, children of both ages helped more in the instrumental condition than in the empathetic condition, and significantly more often in the empathetic condition than in the altruistic condition. This suggests that children’s motivation to help is decreased when there is a cost involved, compared to when there is no cost, as children were less inclined to sacrifice their own possessions in order to help the adult.

The above findings are in line with suggestions by political and theoretical economists that individuals are predominantly inclined to act with informed self-interest, where it is considered to be more profitable to be cooperative in the long-term but selfish in the short-term (e.g., Simon, 1956; Monroe, 1984). However, Hay et al. (1991) argue that this rational approach to resource sharing is in conflict with children’s need to interact positively and harmoniously with others (cf., Paulus and Moore, 2012). In Svetlova et al. (2010) children had to give up something already in their possession, where the pull to self-interest is likely to be at it’s strongest. If young children are indeed driven by self-interest then altruism should remain evident even if the obvious profitability of being selfish in the short-term is reduced and the opportunity to interact positively is increased.

To evaluate this the current experiment implemented a costly helping task whereby young children needed to forgo the opportunity to get a desirable item (i.e., before they had possessed it) in order to help a relative stranger get that item. Specifically, in the primary experimental condition children were presented with an opportunity to help a confederate adult obtain a desirable food reward, an opportunity that would subsequently be made available to the child if she/he chose not to help. Children’s responses in this condition were compared to children who were directly rewarded for helping and to those for whom no direct cost or benefit was made apparent. Based on past findings that children are driven by short-term self-interest we hypothesized that when given an opportunity to help an unfamiliar adult when a future cost is at stake children would be less likely to do so (and slower when they did provide help) than when there was no cost involved, or when there was a direct benefit on offer.

## MATERIALS AND METHODS

### PARTICIPANTS

Forty-five children (19 male and 26 female) aged between 3 years 9 months and 4 years 5 months (*M* = 4 years, *SD* = 7.24) participated in this study, which took place at dedicated testing facilities of a large, metropolitan university. We chose to study children at this age as prior research has documented 3- to 4-year-olds behave selfishly when they must make a choice between a division of resources (e.g., Thompson et al., 1998; Fehr et al., 2008; Paulus and Moore, 2014; Williams and Moore, 2014). Participants were recruited through a database of parents who had previously expressed interest in having their children participate in research. An additional child was omitted from the final sample due to a malfunction with the recording equipment. Of the final sample of 45 children, 44 spoke English as their primary language (the other spoke Japanese, but was bilingual) and the vast majority had parents who had at least 12 years of schooling (95% of mothers; 93% of fathers). Children were allocated in equal numbers to one of three conditions (detailed below). This study was cleared in accordance with the ethical review processes of the University of Queensland and within the guidelines of the National Statement on Ethical Conduct in Human Research.

### MATERIALS

#### Box

**FIGURE 1:**
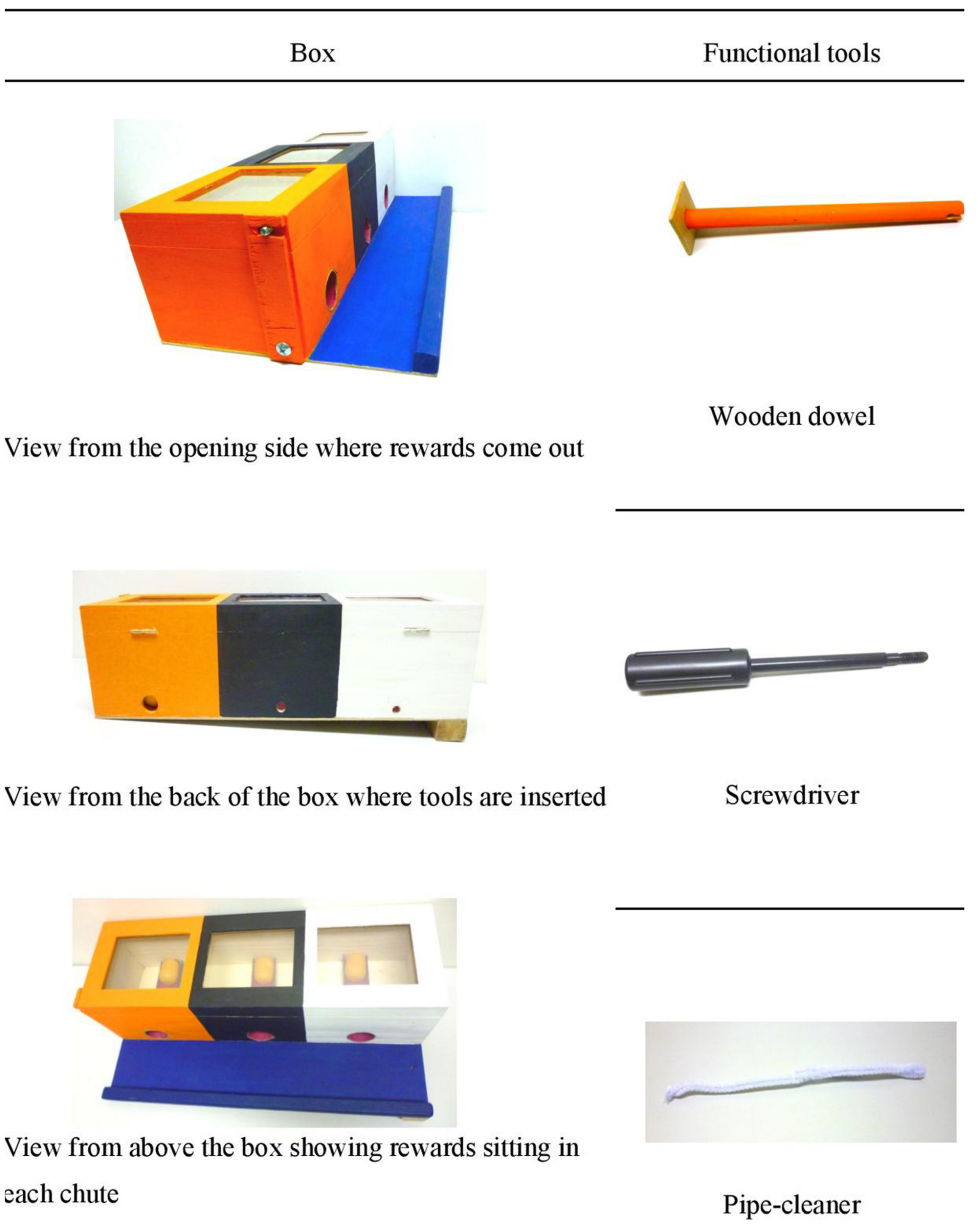
**Test apparatus and associated tools**.

Children were presented with a rectangular wooden box (48.2 cm × 25.5 cm × 13.2 cm) made up of three different colored compartments (see **Figure 1**), mounted on a wooden base. The lid of the box could be fixed shut with a wooden latch, and the lid was transparent, allowing children to see the reward when placed inside. Each reward consisted of a plastic orange pod that contained two jellybeans (or two stickers if parents preferred their children did not receive jellybeans). Each compartment had a different sized opening on one side (1, 1.3, and 2.1 cm diameter, for the white, black, and orange compartments respectively), which lined up with a chute in which the pods were placed. On the other side of each chute were larger openings (4 cm diameter) from which each pod could exit the apparatus.

#### Tools

Three tools (see **Figure 1**) were presented to the children: (1) a 22 cm long orange wooden dowel; (2) a 20 cm long black screwdriver; and (3) a 30 cm long white pipe-cleaner. Each tool was used to retrieve a reward from its corresponding compartment as determined by their matching color.

The three-compartment apparatus was used to provide some independence across trials while minimizing the need for children to learn and remember how to operate each component. The explicit tool-compartment matching was done to ease the cognitive load of the task by enabling children to link the appropriate tool to the appropriate section of the apparatus.

### PROCEDURE

On arrival at the university the child and his/her caregiver were brought into a ‘warm up room,’ in which they could play with toys and become familiar with the two experimenters (E1 and E2) and the test-environment. Once children seemed comfortable and at ease, they were brought into the test room by E1. The children and E1 sat on the floor of the test room with the box and three tools placed between them. The child’s caregiver(s) also sat in the room and was given two questionnaires to complete, so that his/her attention was taken away from the child at test. During the entire procedure the box was only ever loaded with one pod at a time, with the order in which each compartment was used counterbalanced across children and conditions. E1 first showed the child how to retrieve the pod using the relevant tool, and then gave him/her a turn. All children were successfully able to use the tools to retrieve the appropriate pod. Following the child’s turn a confederate (E2), who was blind to the study aims and hypotheses, entered the room where she ‘tried’ to retrieve the pod but to no avail. That is, she deliberately exhibited actions that were not functional in retrieving the pod, such as using her fingers to try and push the pod out or using the wrong tool. This procedure was repeated three times, so the children had an opportunity to help E2 retrieve the pod from each compartment. The specific order of events was as follows:

E1 attracted the participants’ attention to the reward inside the box by saying “Can you see the orange pod inside there? That has two jellybeans inside it. I’ll show you how to get it out.” E1 then demonstrated how to retrieve the reward using the appropriate tool. For example, when demonstrating how to retrieve the reward from the orange compartment, E1 picked up the orange dowel, slid it in the hole and pushed the reward out the other side. While doing this action E1 stated: “For this compartment, we need to use the orange stick … look! We can push the pod out like this.” When the pod came out of the box, E1 opened it to show the jellybeans inside. E1 then placed the pod back into its compartment (surreptitiously opening and closing the lid by disengaging and re-engaging the latch out of the child’s sight) and let the participants have a turn at retrieving it, saying: “It’s your turn now, you get the pod out.” Once the participants retrieved the pod, E1 placed it back in its compartment and said “Ok now [E2’s name] is going to come in and she is going to have a turn at getting the jellybeans out. She has never seen this box before.” E1 then opened the door for E2 who subsequently sat opposite the child. E1 proceeded to show E2 the reward in the box, saying: “Do you see that pod? It has two jellybeans inside. See if you can get it out.” What was said next was determined by which of the following three conditions participants were randomly allocated to:

*Cost condition.* E1 said to the child, “If [E2’s name] gets the pod out, then she gets to have the jellybeans! But if she *can’t* get it out, then you can have another turn and if you get the pod out then you can have the jelly beans.” This was termed the “cost” condition because children choosing to help E2 forfeited the jellybeans they could have received when her turn was over.
*Benefit condition.* As with the cost condition E1 said, “If [E2’s name] gets the pod out, then she gets to have the jellybeans.” However, E2 then said to the child, “Well I don’t like jellybeans, so if I get them out then I am going to give them to you.” This set up a beneficial situation as children choosing to help E2 would directly receive any jellybeans obtained.
*No cost condition.* E1 said to the participants, “[E2’s name] is going to have a turn to get the pod out now.” This was a neutral condition where no explicit cost or benefit of helping was outlined.

After providing the condition-dependent explanation of what was happening E1 said to E2: “Ok you can try to get that out now, I just have to go outside and finish some work.” E1 then left the room. This was done to minimize social pressure. E2 was then given 30 s to ‘try’ to retrieve the reward. Across all trials E2 began by alternating eye gaze between the apparatus and the child, with a neutral facial expression. If the child did not spontaneously help she proceeded to poke her fingers into the relevant compartment, periodically looking at the child and shrugging her shoulders as if to indicate ignorance of what to do. At no point did E2 make any statement about what she was trying to do nor did she directly request help from the child.

#### Helping scenario

If the child helped E2 retrieve the pod within 30 s E2’s actions were condition-dependent as follows:

*Cost condition.* E2 took the jellybeans out of the pod and said “Great, I’m going to eat these jellybeans now,” then left the room. This served to highlight how helping would lead to the child forsaking the potential for accessing the reward him/herself.
*Benefit condition.* E2 took the pod, opened it and stated “I don’t like jellybeans remember, so here, you can have these,” then gave the pod with the jellybeans to the participant and left the room.
*No cost condition.* E2 took the pod and opened in to look inside, but did not state that she was going to have the jellybeans, saying “Cool, two jellybeans!” as she left the room.

E1 then returned, re-loaded the box with the second pod in a different compartment, and stated “I have another pod here with two jellybeans inside it, I’m going to put it in the black/white/orange compartment this time.” E1 then invited E2 to reenter the room and they followed the same procedure as per the first pod. This was repeated for the final pod.

#### No help scenario

If participants did not help E2 in the 30 s trial period, E1 returned to the room and E2 said to her, “I couldn’t do it!” and subsequently left the room. If the participant was in the cost condition, E1 then said to him/her, “Ok well you can try and get the pod out now,” and gave the participant another turn, as promised. If the participant was in the no cost or benefit conditions, E1 removed the first pod then re-loaded the box by placing the second pod in another compartment, stating: “I have another pod here with two more jellybeans inside it, I’m going to put it in the black/white/orange compartment this time.” The afore-outlined procedure was followed and then repeated for the third compartment.

As the participant would be losing out on, or gaining, increasing numbers of jellybeans the more help they provided, the procedure was designed in such a way that the cost or benefit of helping the confederate (depending on which condition the participant was in) would become more apparent and intense as the experiment continued. For example, if a child in the cost condition helped the confederate retrieve all three pods he/she would losing out on six jellybeans.

### CODING

Data was scored from videotapes of each session. Each child’s helping behavior (i.e., was help shown or not) for each of the three compartments was coded (hence children could score between 0 and 3 for helping), and how long it took them to begin helping (in seconds), timed from the moment E2 began to operate on the box. A random sample of 12 children was analyzed by a second coder, blind to the study aims and the conditions each child was in. Intra-class correlations (Shrout and Fleiss, 1979) were above 0.98 (*p* < 0.001) for all latency measures and there was 100% agreement regarding helping behavior (i.e., Cohen’s kappa = 1.00, *p* < 0.001).

## RESULTS

### TOTAL HELP PROVIDED

As is evident in **Table 1**, the vast majority of participants (37 out of 45) provided help on all three compartments, regardless of condition. Consistent with this, a one-way ANOVA failed to reveal any significant differences in the amount of total help provided between the Cost (*M* = 2.67, *SD* = 0.72), No Cost (*M* = 2.87, *SD* = 0.35), and Benefit (*M* = 2.47, *SD* = 1.13) conditions, *F*(2,42) = 0.94, *p* = 0.40, $\eta_{p}^{2}$ = 0.04.

**Table 1:** The number of children providing help over three compartments (zero help, helped on 1 compartment, helped on 2 compartments, or helped on all 3 compartments).

Condition	Zero compartments	One compartment	Two compartments	Three compartments
Cost	0	2	1	12
No cost	0	0	2	13
Benefit	2	1	0	12
Total	2	3	3	37


We also examined the time it took participants to help E2 retrieve the reward on the first, second, and third compartment. There was no significant difference between conditions in the time participants took to help the confederate on the first compartment (*M*_Cost_ = 5.83 s, *SD* = 3.35 s; *M*_NoCost_ = 5.46 s, *SD* = 5.68 s; and *M*_Benefit_ = 3.67 s, *SD* = 5.98 s), *F*(2,38) = 0.62, *p* = 0.513, $\eta_{p}^{2}$ = 0.04. There was also no difference for the time taken to help on the second compartment (*M*_Cost_ = 3.63 s, *SD* = 1.94 s; *M*_NoCost_ = 6.54 s, *SD* = 7.94 s; and *M*_Benefit_ = 3.21 s, *SD* = 2.95 s), *F*(2,35) = 1.59, *p* = 0.218, $\eta_{p}^{2}$ = 0.08, or on the third compartment (*M*_Cost_ = 3.88 s, *SD* = 4.24 s; *M*_NoCost_ = 2.83 s, *SD* = 2.70 s; and *M*_Benefit_ = 1.65 s, *SD* = 1.43 s), *F*(2,38) = 1.80, *p* = 0.18, $\eta_{p}^{2}$ = 0.09.

### LATENCY TO HELP ACROSS COMPARTMENTS

Given the lack of any differences, data was collapsed across conditions. A repeated measures ANOVA, including those children who helped on all three compartments, was performed in order to assess the overall differences in the duration for children to provide help at each compartment (the first, second, and third). Results revealed a significant main effect of instance of help provided, *F*(2,70) = 4.12, *p* = 0.02. *Post hoc* paired-samples *t*-tests revealed that the time taken for children to help E2 on the third compartment (*M* = 2.74 s, *SD* = 3.09) was significantly quicker than the time taken to help her on the first compartment (*M* = 4.89 s, *SD* = 5.14), *t*(36) = 2.67, *p* = 0.011, with time taken to help on the second compartment (*M* = 3.57 s, *SD* = 3.17) falling in between the time taken on the first *t*(36) = 1.66, *p* = 0.106, and third compartments, *t*(36) = 1.27, *p* = 0.213.

## DISCUSSION

It has been previously reported that children’s prosocial motivations decrease when the demands of a task involve sacrifice to the helper something already possessed (Svetlova et al., 2010). We thus expected that when given an opportunity to help an unfamiliar adult when a future cost is at stake children would be less likely to do so than when there was no cost involved, or when there was a direct benefit on offer. Contrary to this expectation, the amount of help children provided was at near ceiling levels across all conditions; that is, the large majority of children helped the confederate retrieve the reward from all three compartments. They were no more likely to help or to be quicker doing so when a direct benefit was involved than when there was a direct cost.

Moreover, when the data was collapsed across conditions, it was found that children increased the speed with which they helped the experimenter. One explanation of this could be that that children may have initially had doubts about whether they were allowed to interact with the box or touch any of the tools when it was not their ‘turn.’ Perhaps once realizing that they were welcome to help E2 after doing so for the first time without being reprimanded, they subsequently helped faster for the other two compartments as this doubt in their minds was alleviated. Regardless of the reasons, what is key is that this increase in the speed of helping even occurred in the condition where the total cost incurred also increased with each act of helping.

The overall results of the current experiment would suggest that children are highly other-regarding, even in situations where self-serving motivations may be in direct competition with that of others. These results contrast with previous experimental studies which report that even when children do provide help when it is costly to them, their helping behavior is more delayed as their motivation to help is lessened by the threat to their own welfare (Svetlova et al., 2010). Moreover, it has been argued that clear communicative cues are necessary for early prosocial responding to occur, and are important for young children to understand others desires (Brownell et al., 2009; Svetlova et al., 2010). However, the children in our study provided spontaneous help in the absence of explicit instruction or verbal cues. This suggests that, at least by around 4 years of age, explicit communicative cues are not needed to elicit helping.

Nevertheless, we cannot know for certain if children were truly acting on their prosocial motivations, or if they were perhaps just imitating prosocial behaviors that they understood to be appropriate for the situation. Williamson et al. (2013) established that by 2 years of age, through observing others, children can learn and apply the appropriate behavioral solution for a specific situation. So it may be possible that by 4 years of age, children have seen others require help, and learnt that the appropriate behavior in that situation is to assist the individual in need: and hence their responses may be considered more normative than altruistic. Future research is needed to evaluate this possibility.

Regardless of the reasons for their behavior, the current study provides an interesting insight into 4-year-olds prosocial tendencies, demonstrating that they spontaneously act to aid a stranger in need. However the study is not without its limitations. Parent presence in the testing room was unavoidable as often both children and parents wished to stay together for the duration of the experiment. Despite the experimenters providing parents with a questionnaire to complete while in the room in order to divert their attention away from their child, parents could still watch their children and their mere presence may been enough to cause an increase in helping behavior. Further, jellybeans are an attractive reward for young children and we chose to provide only a small number as a way of emphasizing their scarcity. It is nevertheless possible that if the attractiveness of the reward is increased children’s tendency to forego them will decrease, as has been shown to be the case in older children (Sierksma et al., 2014). These are matters for future research. Finally, it is possible children simply failed to appreciate the penalty inherent in the cost condition. While this may be true it seems unlikely given the lack of shift in behavior from the first to the third trials. If children did not recognize the cost involved in helping E2 on the first trial surely they would have by the third. Yet there was no discernable change in helping across trials.

A key feature of the task used here is that children needed to act in the present while taking into consideration future possible outcomes. Perhaps the high level of apparent altruism revealed here is primarily a reflection of an immature capacity for doing this. We did not evaluate episodic foresight abilities in the children we tested. However, past studies have established that by 4 years of age children can import a past event from long-term memory into working memory and act for the future (Suddendorf et al., 2011; Redshaw and Suddendorf, 2013; Suddendorf and Redshaw, 2013; Prabhakar and Hudson, 2014; Atance et al., 2015). Thus, while possible, it is unlikely that a deficit in episodic foresight accounts for the behavior of the children documented here.

A strength of the current study is that where much of the previous research examining altruistic helping in young children has required children to make a discrete choice between helping when it is costly or not helping at all (e.g., Brownell et al., 2009; Moore, 2009), the current experiment allows children’s decisions to be more flexible in that they could choose to help, and only to a certain extent; that is they could help retrieve just one reward and so incur a minimal cost, or help retrieve all three reward and incur a larger cost. This method reflects situations whereby children may choose to help on a continuum, and hence we believe it is an ecologically valid method of assessing children’s prosocial tendencies.

The current study also represents a departure from prior work in which children must make a choice between a division of resources that is self-advantageous, neutral or other-advantageous (e.g., Thompson et al., 1998; Fehr et al., 2008; Paulus and Moore, 2014; Williams and Moore, 2014). In these studies children, especially in the age band tested here, eschew sharing resources, although some will make choices that benefit others if costs to the self are minimal. Why, then, were children so willing to forego reward in the current experiment? A possible explanation is that in the afore-cited studies emphasis is placed on sharing and appraisal of inequity, whereas here emphasis is on helping and behaving prosocially. A shift in emphasis of this nature may be enough to flip behavior. This calls for future research to explore whether preschool children will show a heightened willingness to share at a cost if such sharing is framed as a helping endeavor.

Conversely, research undertaken in the last decade has established young children’s proclivity to copy the actions of others, including actions that are clearly irrelevant to the demonstrated, functional outcome (e.g., using a stick to open a box in order to retrieve a toy after first wiping the stick across the box’s lid), a proclivity that the available literature suggests is species-specific, culturally universal and likely to increase in intensity with age (Horner and Whiten, 2005; Nielsen and Tomaselli, 2010; McGuigan et al., 2011; Flynn and Smith, 2012; Nielsen et al., 2012, 2014; Marsh et al., 2014). In a recent study, children were shown how to open a box by an adult who used a sequence of actions, some which were causally relevant and some which were not (Nielsen et al., in press). The children could then show an ostensibly naïve individual how to open the box while the first experimenter was absent. Even under these circumstances children reproduced the redundant actions. A similar behavior might be happening in the current study: that is, children are simply copying the actions of E1, even when only E2 is present. We cannot rule out this interpretation – but if it is valid, and future research is needed to evaluate this possibility, it would suggest children afford less priority to getting a treat for themselves than they do showing they have acquired a new skill or that they can do things as others have done them. This would stand as a major signifier of our status as the world’s most “ultra-social” species (Herrmann et al., 2007, p. 1360).

In this context, a number of authors have argued that children copy others in order to identify and affiliate with them (e.g., to be liked by them or to show that “I am like you” ; Uzgiris, 1981; Nadel et al., 1999; Nielsen, 2008; Carpenter, 2010; Nielsen and Blank, 2011; Over and Carpenter, 2012). This perspective mirrors the view that (some) prosocial behaviors are driven by children’s motivation for interaction and not by a genuine motivation to do something to help others (Paulus and Moore, 2012; see also Hay, 2009). This interpretation fits the data we present here, and indeed is more aligned with it than views of the emergence of prosocial behavior that emphasize empathic concern for the needs of others (Batson, 1991; Hoffman, 2000), acting to either alleviate shared distress (Kärtner et al., 2010), or acting on behalf of another having interpreted his/her goals as if they were one’s own (Kenward and Gredebäck, 2013). However, as already noted, the presence of parents in the test room may have influenced children’s reactions, something that would be consistent with social-normative models prioritizing the role of the social environment and social inputs in the emergence of prosociality (Kiang et al., 2004; Brownell et al., 2009). It is also possible that the behavior of different children is determined by different motivations, and as Paulus (2014, p. 79) notes in a recent review, “it seems unlikely that the domain of prosocial behavior as a whole is brought about by one mechanism or motive.”

The current study provides new insight into the altruistic behavior of young children. Children may be motivated to help others because doing so creates a reputation that will be rewarded in the future (e.g., Trivers, 1971), as part of an inherent drive to maintain sociality regardless of confronting environmental events (e.g., Gintis, 2000), because their capacity for empathy drives them to assist others, or for some hitherto unidentified other reason. Regardless of why, this motivation appears to be a strong one. Continued research is now needed to determine how strong: research that will help delineate our understanding of what may prove to be one of the key features of the human mind.

## Conflict of Interest Statement

The authors declare that the research was conducted in the absence of any commercial or financial relationships that could be construed as a potential conflict of interest.
